# Identification of a metabolism-related gene expression prognostic model in endometrial carcinoma patients

**DOI:** 10.1186/s12885-020-07345-8

**Published:** 2020-09-07

**Authors:** Pinping Jiang, Wei Sun, Ningmei Shen, Xiaohao Huang, Shilong Fu

**Affiliations:** Department of Gynecology, The First Affiliated Hospital of Nanjing Medical University, Nanjing, 210029 Jiangsu Province China

**Keywords:** Metabolism, TCGA, Endometrial carcinoma, Prognostic model, Nomogram

## Abstract

**Background:**

Metabolic abnormalities have recently been widely studied in various cancer types. This study aims to explore the expression profiles of metabolism-related genes (MRGs) in endometrial cancer (EC).

**Methods:**

We analyzed the expression of MRGs using The Cancer Genome Atlas (TCGA) data to screen differentially expressed MRGs (DE-MRGs) significantly correlated with EC patient prognosis. Functional pathway enrichment analysis of the DE-MRGs was performed. LASSO and Cox regression analyses were performed to select MRGs closely related to EC patient outcomes. A prognostic signature was developed, and the efficacy was validated in part of and the entire TCGA EC cohort. Moreover, we developed a comprehensive nomogram including the risk model and clinical features to predict EC patients’ survival probability.

**Results:**

Forty-seven DE-MRGs were significantly correlated with EC patient prognosis. Functional enrichment analysis showed that these MRGs were highly enriched in amino acid, glycolysis, and glycerophospholipid metabolism. Nine MRGs were found to be closely related to EC patient outcomes: CYP4F3, CEL, GPAT3, LYPLA2, HNMT, PHGDH, CKM, UCK2 and ACACB. Based on these nine DE-MRGs, we developed a prognostic signature, and its efficacy in part of and the entire TCGA EC cohort was validated. The nine-MRG signature was independent of other clinical features, and could effectively distinguish high- and low-risk EC patients and predict patient OS. The nomogram showed excellent consistency between the predictions and actual survival observations.

**Conclusions:**

The MRG prognostic model and the comprehensive nomogram could guide precise outcome prediction and rational therapy selection in clinical practice.

## Background

Endometrial carcinoma (EC), one of the most common female reproductive malignancies, caused nearly 90,000 deaths worldwide each year [1]. Women with metabolic disorders, including obesity and diabetes, have a markedly increased risk of developing endometrial cancer. While early-stage endometrial cancer has a favorable prognosis, nearly 30% of patients are still diagnosed at a late stage, and over 80% of these individuals die in 5 years [2]. In addition, several EC patients present a high risk of cancer progression or recurrence with insensitivity to chemotherapy, which indicates poor outcomes [3]. Therefore, it is imperative to emphasize the molecular changes that occur during endometrial cancer progression and develop novel predictive biomarkers to accurately estimate patient outcomes.

Since fundamental metabolic differences between cancer and adjacent normal cells were first uncovered, metabolic reprogramming has increasingly become a hot topic in cancer biology [4]. The metabolic phenotype of cancer cells is heterogeneous in various cancer types; for example, while several malignant tumors mainly rely on glycolysis, others present a metabolic phenotype mediated by oxidative phosphorylation [5, 6]. Overall, through reprogramming tumor microenvironments, catabolic and anabolic metabolism is essential for cancer cells to sustain energy supply and biomass synthesis [7–9]. While the underlying processes and molecular alterations of metabolic programming in various cancers have been well elucidated, the expression patterns of metabolism-related genes in endometrial cancer are still unclear.

In this study, we focused on the metabolism-related gene expression alterations of The Cancer Genome Atlas (TCGA) EC patients and obtained prognostic dysregulated MRGs. In addition, we established and validated a multiple-MRG-combined expression signature for predicting EC patient outcomes. Moreover, we integrated the clinical features of patients and the MRG model to establish a novel nomogram model that could guide comprehensive EC therapeutic strategies.

## Methods

### Integration of gene expression profiles and clinical information

We downloaded the mRNA expression profiles of EC patients (in the FPKM format) from The Cancer Genome Atlas (TCGA) database (https://tcga-data.nci.nih.gov/tcga/), which contains a total of 541 cases. The cBioPortal for Cancer Genomics (http://cbioportal.org) enables researchers to explore, visualize, and analyze multi-dimensional cancer genomics data and clinical information. The corresponding clinical data of EC was retrieved from the cBioPortal [10].

### Extraction of metabolism-related genes from the TCGA database

Genes enriched in metabolic pathways in the KEGG database were utilized in this study as metabolic genes (Supplementary Table 1) [11]. The mRNA expression of metabolic genes in the TCGA database was extracted.

### Identification of prognosis-associated differentially expressed metabolism-related genes (DE-MRGs)

With the cut-off criteria set as |logFC| > 1 and *P*-value < 0.05, we screened the DE-MRGs via the “limma”R package [12]. Then, univariate Cox regression analysis was performed to identify prognosis-associated DE-MRGs. Hazard ratio (HR) < 1 indicates better overall survival (OS) outcomes while HR > 1 indicates worse OS outcomes. Genes with *P* < 0.05 were regarded as prognosis-associated metabolic genes. The expression levels of the prognosis-associated DE-MRGs in each patient and between cancerous and normal samples were displayed via the “pheatmap” and “ggplot” R packages, respectively.

### Functional enrichment analysis of the prognosis-associated DE-MRGs

Gene ontology (GO) [13] and Kyoto Encyclopedia of Genes and Genomes (KEGG) [11] pathway enrichment analyses were performed to explore the biological functions of the prognosis-related DE-MRGs via the “clusterProfiler” R package. Adjusted *P*-value < 0.05 was set as the significance threshold, and the enrichment analysis result maps were presented by the “ggplot2” and “GOplot” R packages.

### Protein-protein interaction (PPI) network construction and hub DE-MRG alteration analysis

The Search Tool for the Retrieval of Interacting Genes/Proteins (STRING, https://string-db.org/) database comprises the interaction information of given proteins [14]. Based on the minimum required interaction score setting of 0.4, we utilized the STRING database to construct a PPI network, which reflected the interactions among the DE-MRG proteins. The network was visualized by Cytoscape software and the top 15 hub proteins were selected based on their connectivity degree in the PPI network [15]. In addition, the alteration landscapes of the hub DE-MRGs in EC were visualized by cBioPortal.

### Establishment of a prognostic model based on the DE-MRGs

We randomly classified all TCGA EC patients into training and testing cohorts. The least absolute shrinkage and selection operator (LASSO) and multivariate Cox regression analysis were then performed to select key prognosis-related DE-MRGs via the “glmnet” R package. The formula of the risk score for the prediction of EC patients’ prognosis was as follows: risk score = the sum of the multivariate Cox regression coefficient ratio of each mRNA multiplied by the expression level of each mRNA. Based on the median risk score, we divided the training cohort patients into high- and low-risk subgroups. In the two subgroups, each patient’s survival status, OS time, and gene expression profile were presented via the “pheatmap” and “survival” R packages. In addition, the Kaplan-Meier curve analysis was performed, and receiver operating characteristic (ROC) curves were drawn to estimate the sensitivity and specificity of the prognostic signature.

### Validation of the efficacy of the prognostic DE-MRG signature

The prognostic signature was then introduced into the testing cohort and the entire cohort. Based on the median risk score from the training cohort, the patients in the testing cohort and entire cohort were separated into high- or low-risk groups. Kaplan–Meier curve analysis, time-dependent ROC analysis, and patient outcome distribution were performed.

### RNA extraction of clinical samples and quantitative real-time RT-PCR (qRT-PCR) analysis

A total of 30 RNA later-reserved EC specimens were collected from patients who underwent surgery at Jiangsu Province Women and Children Health Hospital (Nanjing, China) between September 2017 and September 2019. All samples were immediately snap frozen in liquid nitrogen and stored at − 80 °C until further analysis. Total RNA was isolated from fresh-frozen tissues using TRIzol reagent (Invitrogen) from fresh-frozen tissues and transcribed into cDNA using a TaqMan Reverse Transcription kit (Applied Biosystems) with random hexamer primers. qRT-PCR was performed using 2 × SYBR Green qPCR Master Mix (Selleck, Shanghai, China). The housekeeping gene GAPDH was used for normalization of the qRT-PCR data before calculation using the ΔΔCt method and Student’s t-test (two-tailed) was used for the comparison analyses. The primers used are listed in Supplementary Table 2.

### Evaluation of clinical independence and construction of the nomogram

Next, we removed EC patients who lacked detailed clinicopathological information including survival status and time, age, weight, clinical stage, tumor grade, and lymph node status. The clinicopathological characteristics and the MRG expression data of the remaining patients were compared between the high- and low-risk subgroups and comprehensively displayed in the heatmap. Moreover, the clinical indexes and risk scores were included in univariate and multivariate Cox regression analyses to validate the independence of the risk model. ROC curves for the signature and other clinical features were used to assess the predictive efficacy of the model. In addition, the correlation between the MRGs from the risk model and the clinical index was also measured. Finally, we utilized the “rms” R package to consolidate the risk score and clinical characteristics for nomogram construction.

## Results

### Identification of prognosis-related DE-MRGs

**Fig. 1:**
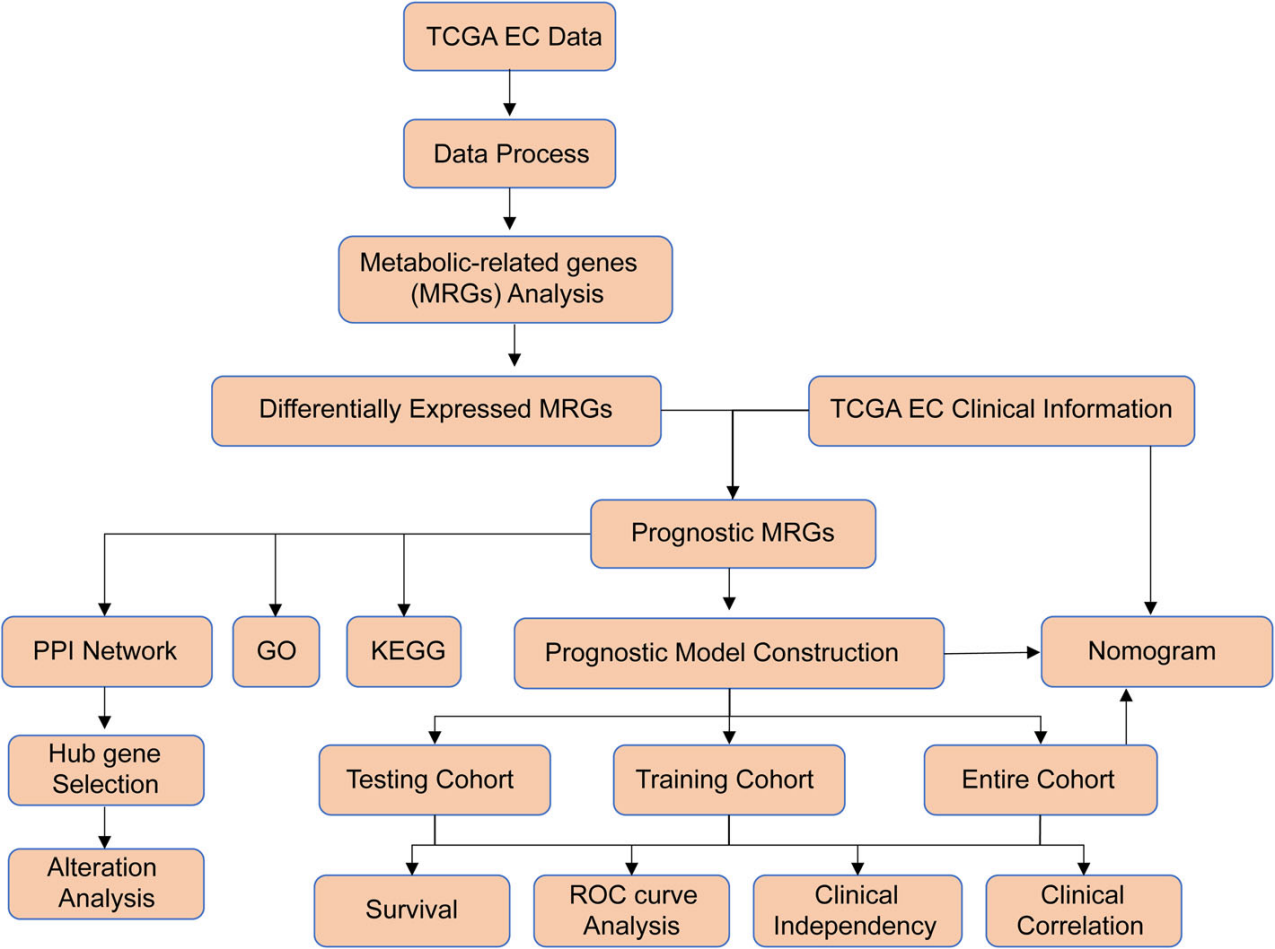
The flow chart of the analysis procedure in identifying a metabolism-related prognostic signature

**Fig. 2:**
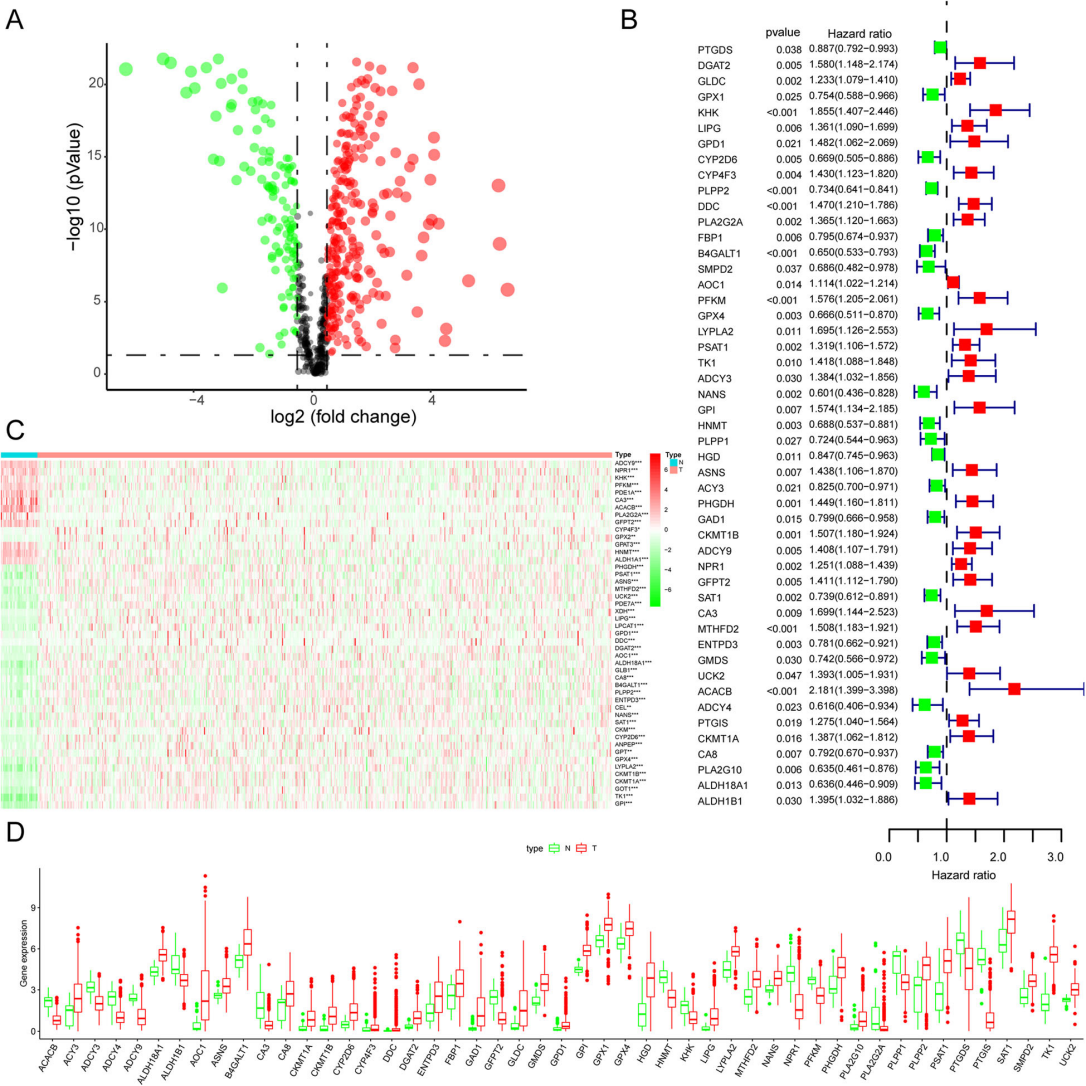
The expression profiles of prognosis-associated differentially expressed metabolism-related genes (DE-MRGs) between TCGA endometrial cancer (EC) and normal tissues. **a** Volcano plot of DE-MRGs in EC and normal samples of the TCGA dataset. The vertical axis indicates the -log(*P*-value), and the horizontal axis indicates the log2 (fold change [FC]). The red dots and the green dots represent up- and down-regulated genes, respectively (*P*-value< 0.05 and |log2(FC)| > 1). **b** Univariate Cox regression identified 47 DE-MRGs correlated to EC patient outcomes; **c** Heat map of the 47 DE-MRGs in the entire TCGA EC cohort. Red and green indicate higher expression and lower expression, respectively. **d** Box plot of the expression of the DE-MRGs between cancerous and normal tissues

The detailed flow chart for the prognostic predictive model construction in this study is shown in Fig. 1. From the metabolic pathways in the KEGG database, we extracted a total of 944 metabolism-related genes. After the integration of the MRG expression data of 552 TCGA EC cancerous and 35 nontumor samples, we obtained 156 upregulated and 64 downregulated MRGs (Fig. 2a). Univariate Cox regression analysis identified 47 genes significantly correlated with EC patients’ OS (Fig. 2b and Table 1). The expression pattern of 47 prognosis-related MRGs is shown in the heatmap and box plot in Fig. 2c-d.

**Table 1:** Univariate Cox regression identified 20 MR-DEGs correlated to endometrial cancer patient OS

Gene ID	HR	HR.95 L	HR.95H	***P***-value
CYP4F3	1.19013465	1.08676961	1.303330965	0.000173367
PHGDH	1.02042871	1.009021493	1.031964888	0.000422248
GPX2	1.002979902	1.001288036	1.004674627	0.000551669
CKMT1B	1.223457962	1.083264863	1.381794458	0.001162169
CEL	1.008855015	1.003431253	1.014308094	0.001348837
DDC	1.098684916	1.0372027	1.16381161	0.001359205
KHK	1.189854228	1.067798895	1.325861162	0.001644451
LYPLA2	1.019470527	1.006509657	1.032598294	0.003137784
ACACB	1.834694401	1.218329674	2.762883985	0.003667941
ASNS	1.052124561	1.015433759	1.090141116	0.00502135
GPAT3	1.104677229	1.028736495	1.186223863	0.006151058
CKMT1A	1.256355384	1.059829197	1.489323803	0.008551614
CKM	1.141628713	1.031351441	1.263697383	0.01060172
UCK2	1.035172213	1.007976028	1.063102178	0.010933586
HNMT	0.861666359	0.760829865	0.975867206	0.019044088
SAT1	0.997992548	0.996252269	0.999735867	0.024031622
B4GALT1	0.995107725	0.990877644	0.999355864	0.024044249
GLB1	0.971544079	0.946835378	0.996897579	0.028065649
PSAT1	1.007191289	1.000533596	1.013893283	0.034207771
PFKM	1.087927137	1.004895375	1.177819587	0.037477496

Abbreviations: *MR-DEGs* metabolism-related differentially expressed genes, *OS* overall survival, *HR* Hazard ratio

### Functional enrichment of the prognosis-related DE-MRGs

**Fig. 3:**
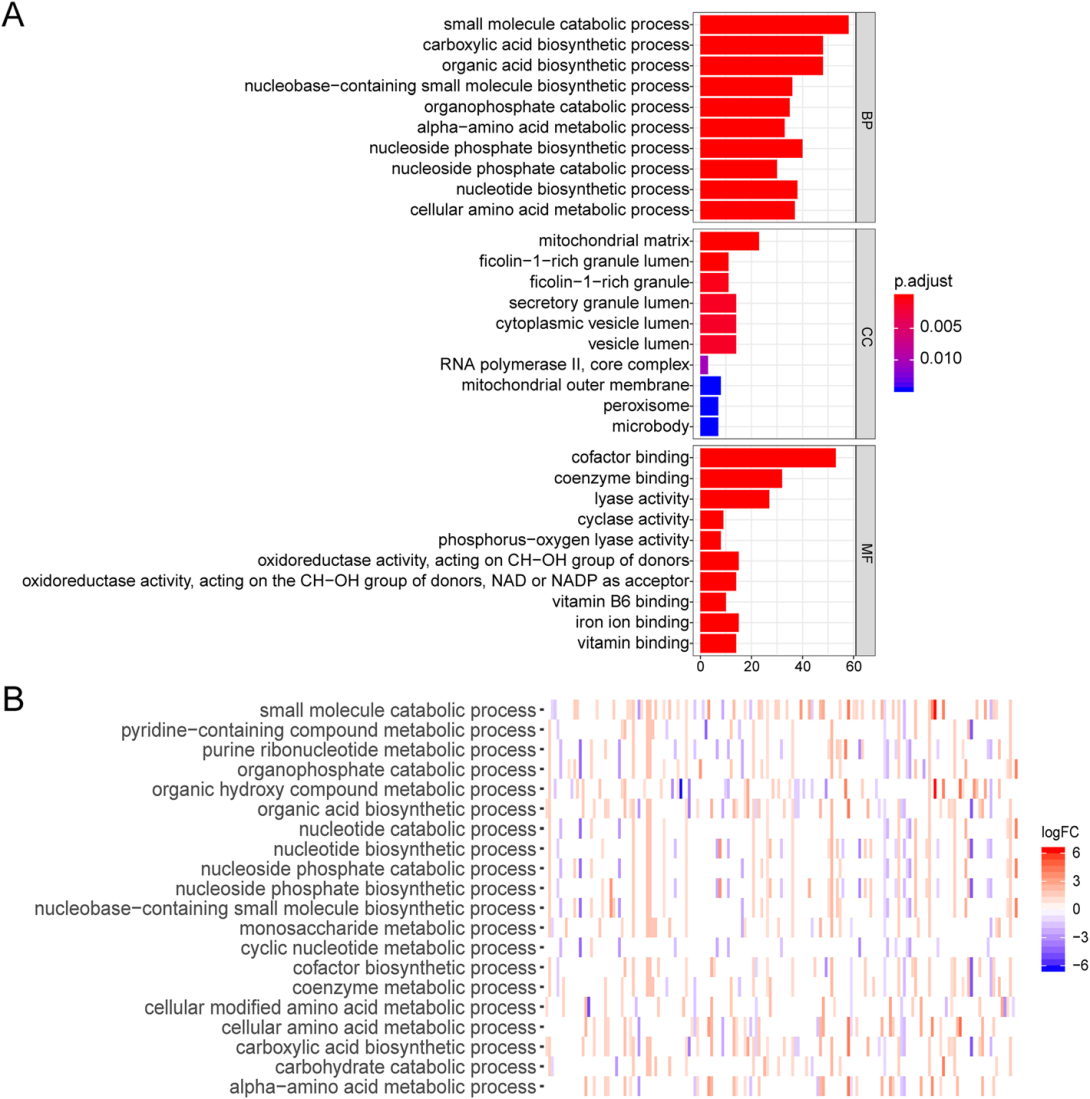
Gene Ontology (GO) functional enrichment of prognostic DE-MRGs. **a** GO analysis shows the biological processes, cellular component, and molecular functions involved in differential genes. **b** Heatmap of the expression of DEGs in the enriched GO items

**Fig. 4:**
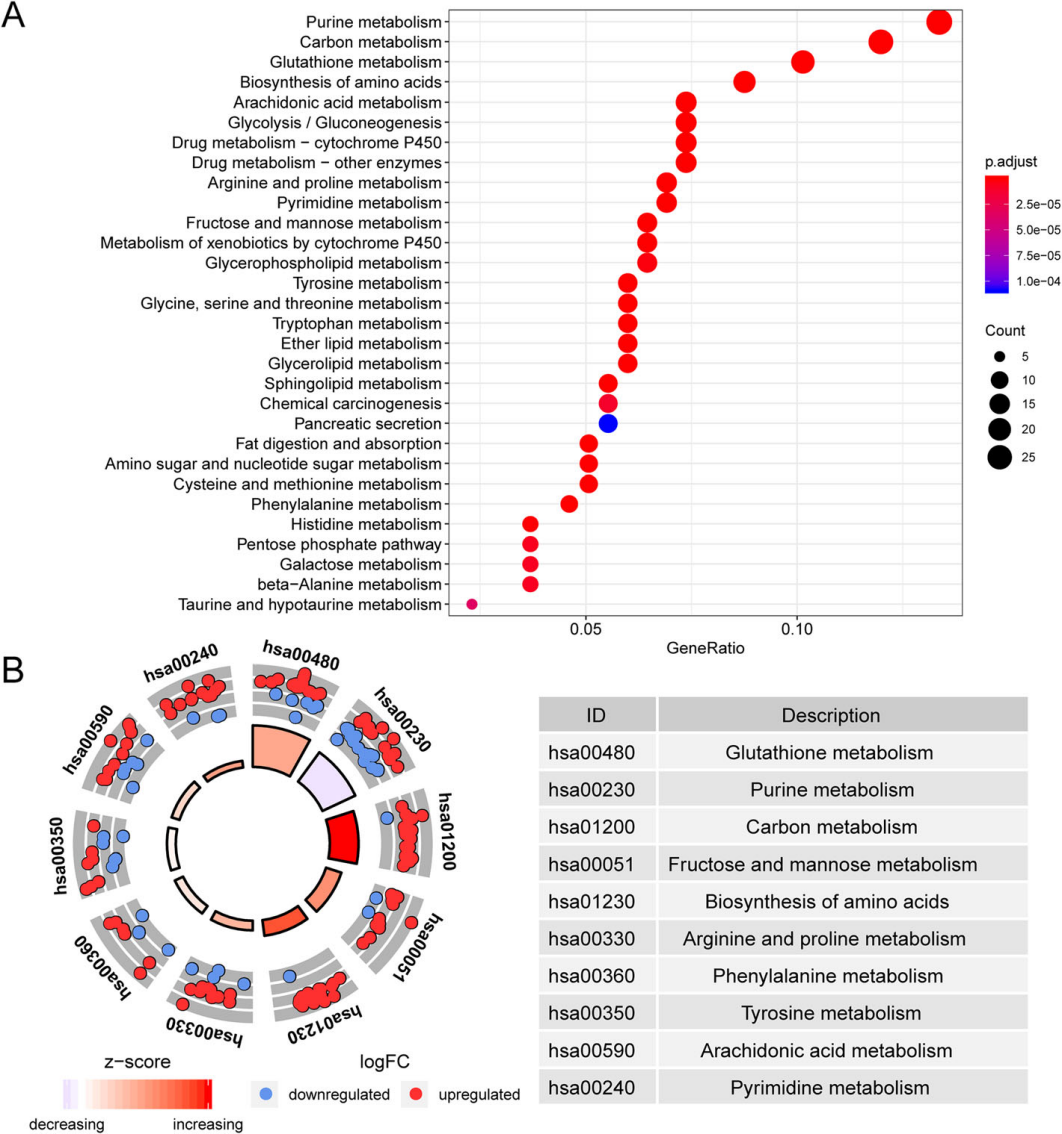
Kyoto Encyclopedia of Genes and Genomes (KEGG) pathways enrichment of prognosis-associated DE-MRGs. **a** KEGG analysis shows significantly enriched pathways of DE-MRGs. The node color changes gradually from red to blue in descending order according to the adjusted *P*-values. The size of the node represents the number of counts. **b** Circle plot of the enriched DEGs in the KEGG items

Function annotation analyses of the 220 DE-MRGs and 47 prognostic DE-MRGs were then performed (Supplementary Fig. 1 and Fig. 3). GO enrichment showed that the prognostic MRGs were mainly involved in “carboxylic acid biosynthetic process “, “cofactor binding “, “alpha-amino acid metabolic process “ and “cellular amino acid metabolic process” (Fig. 3a). The enriched GO terms and related gene expression profiles are presented in Fig. 3b. KEGG pathway enrichment analysis showed that the prognostic DE-MRGs were mainly involved in “purine metabolism”, “biosynthesis of amino acids”, “glycolysis / gluconeogenesis” and “glycerophospholipid metabolism” (Fig. 4a). The expression levels of the correlated genes in the enriched KEGG pathways are displayed in the heatmap (Fig. 4b).

### The PPI network of 47 prognosis-related DE-MRGs and hub gene alteration analysis

**Fig. 5:**
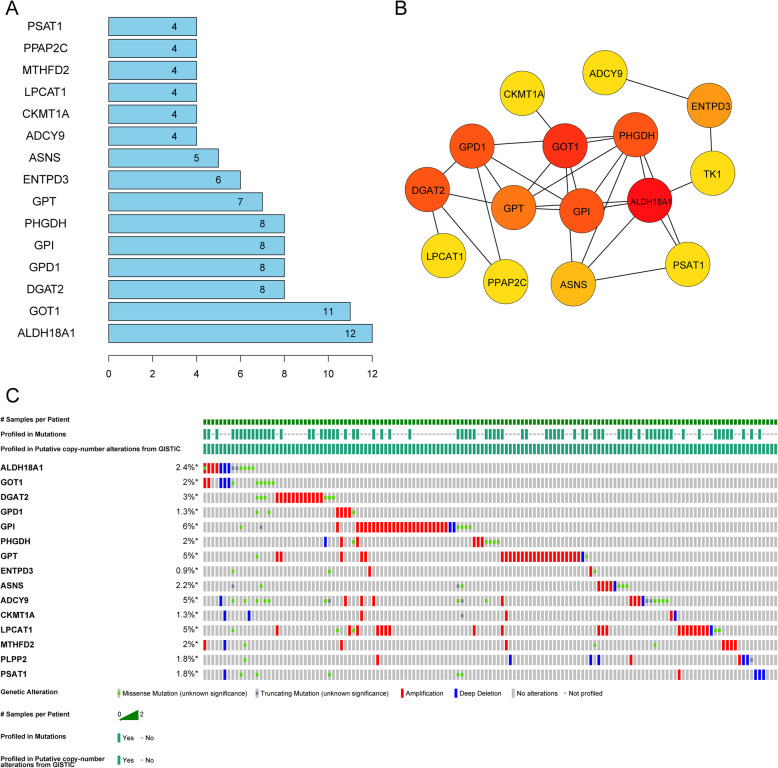
Protein-protein network of the hub DE-MRGs and alteration analysis. **a** The highest degree of hub genes was ranked; **b** The interaction network of the top 15 hub genes; **c** The gene mutation overview of 15 hub genes in TCGA EC patients

Through the STRING website, we built a PPI network of the 47 DE-MRG proteins (Supplementary Fig. 2). There were 47 nodes and 81 edges included in the network based on the interaction score criteria. The top 15 hub proteins with the highest connectivity degrees in the PPI network were as follows: ALDH18A1, GOT1, DGAT2, GPD1, GPI, PHGDH, GPT, ENTPD3, ASNS, ADCY9, CKMT1A, LPCAT1, MTHFD2, PPAP2C and PSAT1 (Fig. 5a-b). The alteration results of the hub genes showed that GPI, GPT, ADCY9, and LPCAT1 ranked as the most frequently altered genes. GPI, GPT, and LPCAT1 were frequently overamplified in endometrial cancer patients, while ADCY9 more often had missense mutations (unknown significance) (Fig. 5c).

### Identification of a nine DE-MRG-based prognostic model

**Fig. 6:**
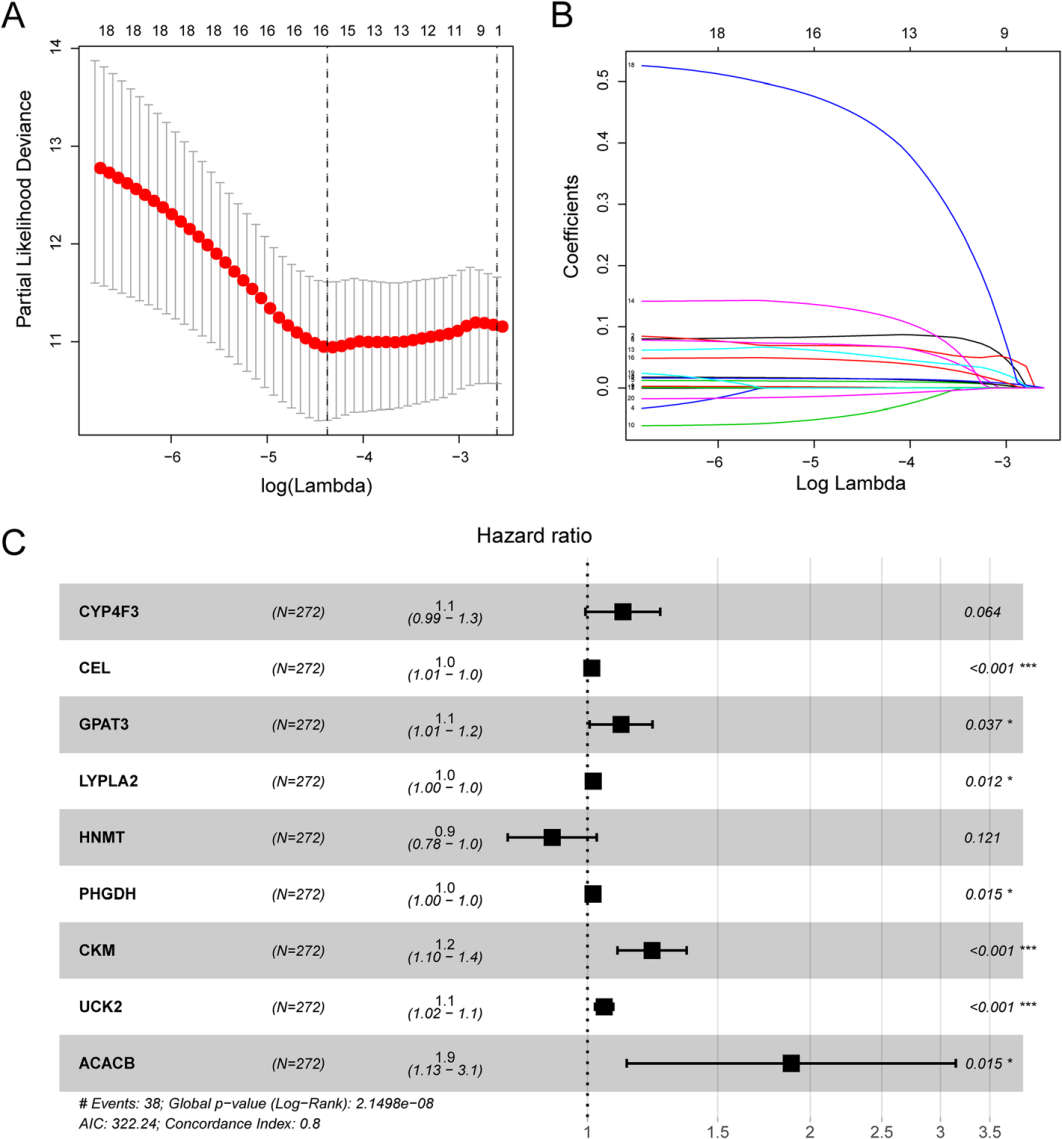
Subsequent identification of prognosis related DE-MRGs using LASSO and Cox regression analysis. **a** Plots of the cross-validation error rates. Each dot represents a lambda value along with error bars to give a confidence interval for the cross-validated error rate; **b** LASSO coefficient profiles of the MRGs associated with the overall survival of endometrial cancer; **c** multivariate Cox regression identified 9 prognostic MRGs in the training cohort

Next, we randomly divided the 541 TCGA EC patients into a training cohort (*n* = 272) and a testing cohort (*n* = 269). LASSO and multivariate Cox regression analyses identified 9 genes significantly associated with prognosis: CYP4F3, CEL, GPAT3, LYPLA2, HNMT, PHGDH, CKM, UCK2 and ACACB (Fig. 6 and Table 2). According to the results of multivariate Cox regression analysis, we constructed the prognostic model as follows: risk score = (0.110103 × expression value of CYP4F3) + (0.013456 × expression value of CEL) + (0.104444× expression value of GPAT3) + (0.017777 × expression value of LYPLA2) + (− 0.10986 × expression value of HNMT) + (0.017183 × expression value of PHGDH) + (0.200964 × expression value of CKM) + (0.051913 × expression value of UCK2) + (0.634313 × expression value of ACACB).

**Table 2:** Multivariate Cox regression selected 9 MR-DEGs correlated to endometrial cancer patient OS

Gene ID	HR	HR.95 L	HR.95H	***P***-value
CYP4F3	1.116392881	0.99351779	1.25446477	0.064219393
CEL	1.013547388	1.007787405	1.019340292	3.69768E-06
GPAT3	1.110093426	1.006471947	1.224383272	0.03670839
LYPLA2	1.017935764	1.003942684	1.03212388	0.011831278
HNMT	0.895961269	0.77990526	1.029287321	0.1206327
PHGDH	1.017331051	1.003306749	1.031551385	0.015262521
CKM	1.222580744	1.097931508	1.36138153	0.00024948
UCK2	1.053283808	1.024064937	1.083336359	0.000298414
ACACB	1.885726048	1.129790043	3.147454476	0.015230463

Abbreviations: *MR-DEGs* metabolism-related differentially expressed genes, *OS* overall survival, *HR* Hazard ratio

**Fig. 7:**
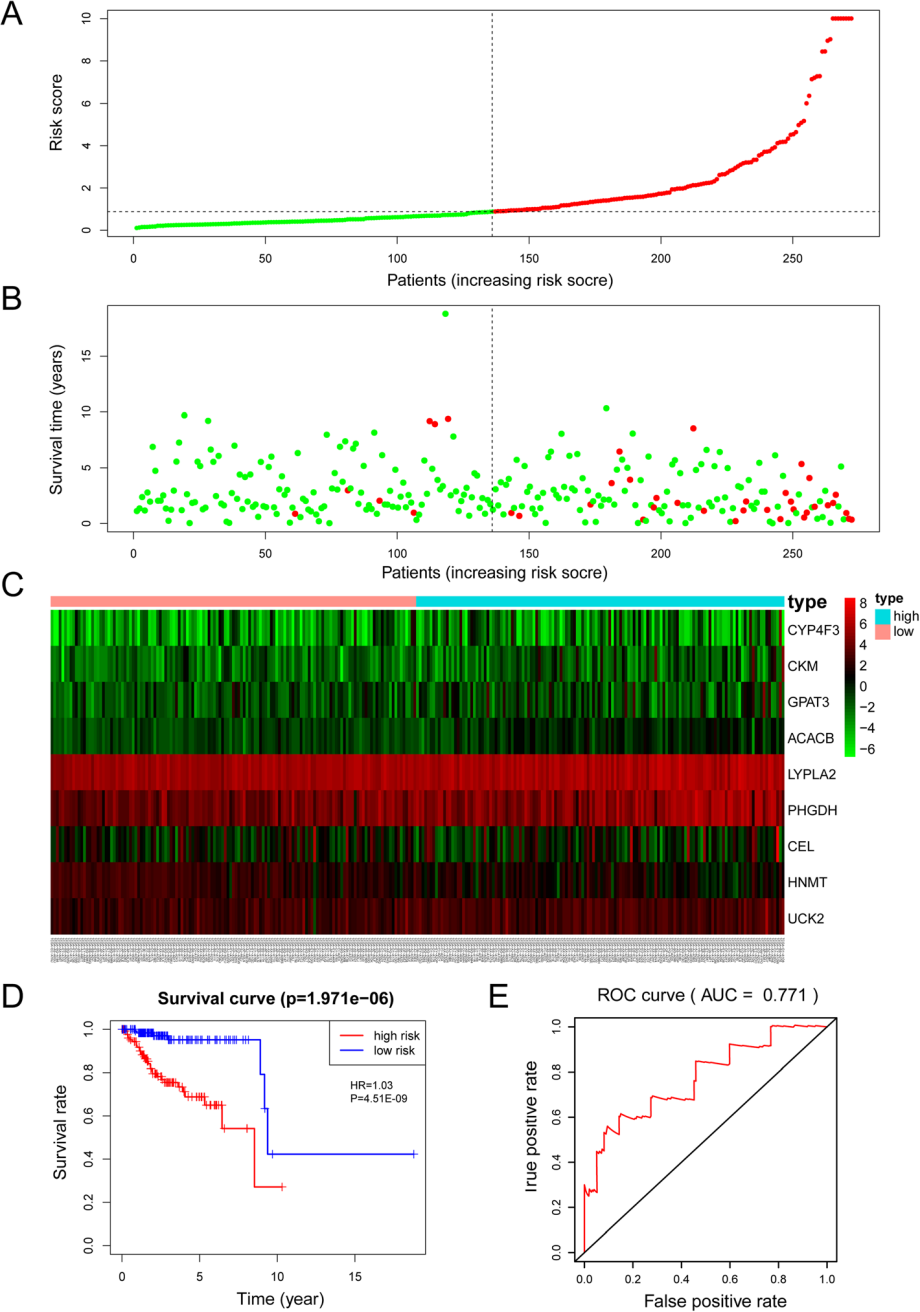
Prognostic analysis of the model in the TCGA training cohort. **a** The risk score, (**b**) survival status, (**c**) expression heatmap, (**d**) Kaplan-Meier survival, and (**e**) time-dependent ROC curves of the prognostic model for the TCGA EC training cohort

Based on the mean risk score from the training cohort, the patients were divided into high-risk (*n* = 136) and low-risk (*n =* 136) subgroups. Each individual’s risk score and survival status were ranked and displayed on the dot plot, which showed significant differences in OS between the groups (Fig. 7a-b). Likewise, the Kaplan-Meier curve analysis demonstrated that the OS of the higher-risk group was significantly shorter than that of the low-risk group (*P* = 1.971e-06) (Fig. 7d). The expression profiles of the 9 prognostic DE-MRGs showed that UCK2, PHGDH, ACACB, LYPLA2, CYP4F3, GPAT3, CEL, and CKM were expressed at higher levels in the high-risk subgroup, while HNMT was expressed at lower levels (Fig. 7c). ROC curve analysis revealed that the area under the ROC curve (AUC) of the prognostic MRG model was 0.771 (Fig. 7e).

### Validation of the efficacy of the 9-MRG prognostic signature

**Fig. 8:**
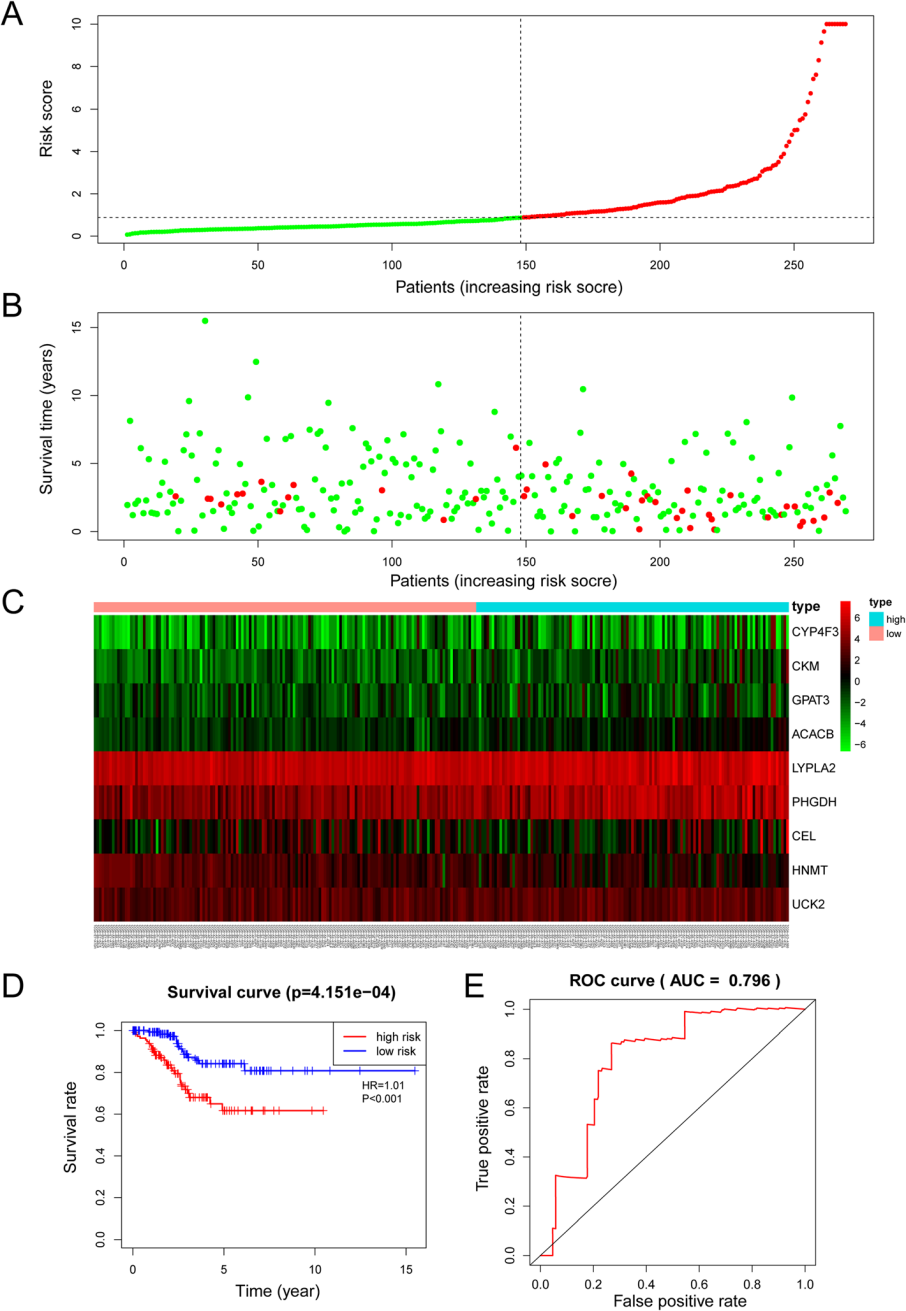
Validation of the efficacy of the risk signature in the TCGA testing cohort. **a** The risk score, (**b**) survival status, (**c**) expression heatmap, (**d**) Kaplan-Meier survival, and (**e**) time-dependent ROC curves of the prognostic model for the TCGA EC testing cohort

**Fig. 9:**
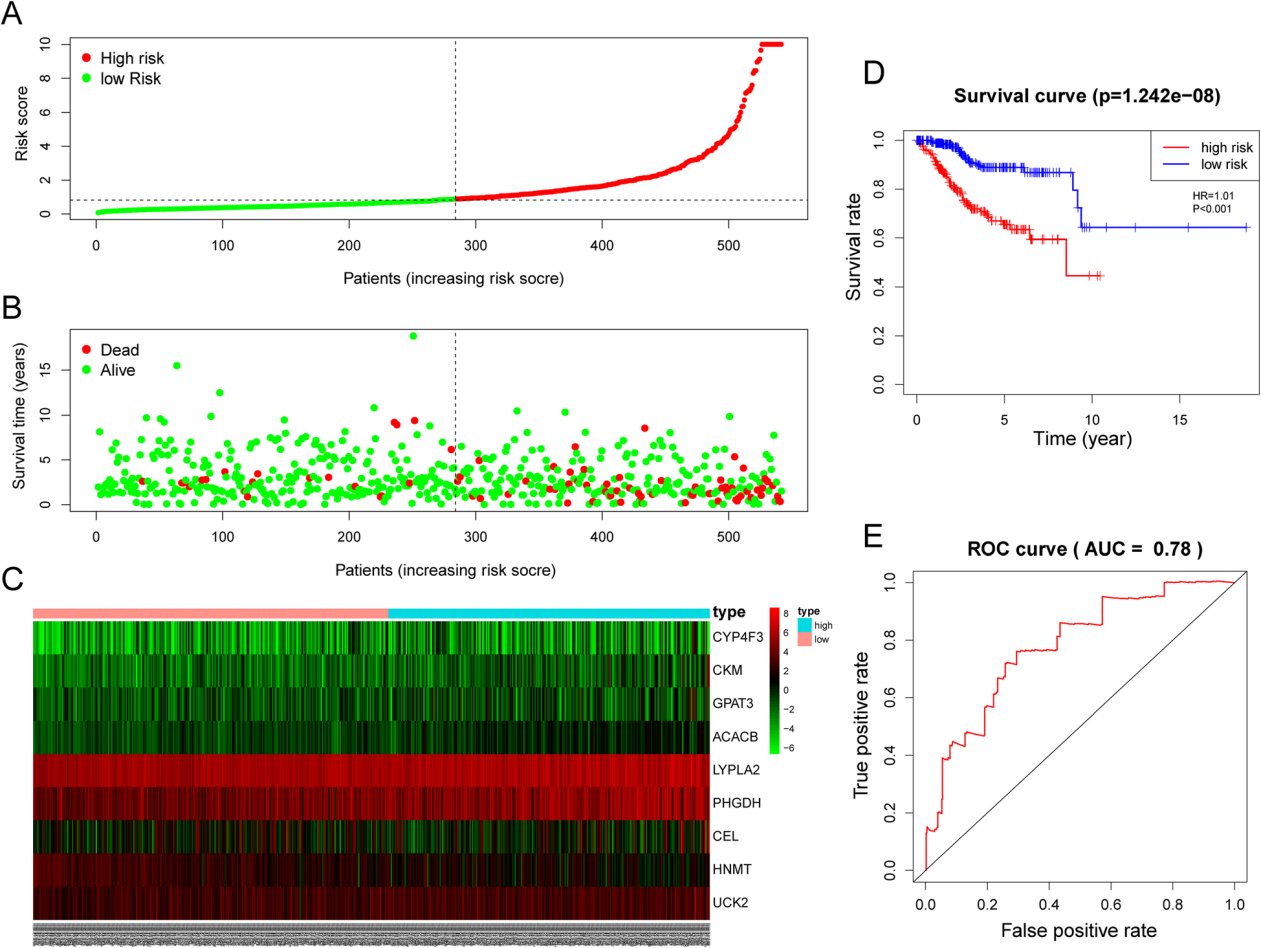
Estimation of the efficacy of the risk model in TCGA entire EC cohort. **a** The risk score, (**b**) survival status, (**c**) expression heatmap, (**d**) Kaplan-Meier survival, and (**e**) time-dependent ROC curves of the prognostic model for the TCGA EC entire cohort

The risk model was then introduced into the testing cohort and entire cohort, and each individual’s risk score was calculated. Based on the training cohort cut-off risk score, the patients in the testing cohort were classified as 121 high-risk and 148 low-risk individuals. In agreement with the results from the training cohort, the survival status (Fig. 8a), survival time (Fig. 8b) and KM curve analysis of the high-risk subgroup presented worse outcomes than those of the low-risk subgroup in the testing cohort with a shorter overall survival time(*P* = 4.151e-04) (Fig. 8d). The expression patterns of the 9 MRGs were consistent with those in the training cohort (Fig. 8c), and the AUC of the risk model in the testing cohort was 0.796 (Fig. 8e). Similar results were also observed for the entire cohort. The low-risk subgroup presented longer survival times and better survival statuses (Fig. 9a-b). In addition, the expression profiles of the 9 MRGs were in line with those in both the training cohort and the testing cohort (Fig. 9c). Kaplan-Meier curve analysis showed that the low-risk subgroup had longer OS times (*P* = 1.242e-08) (Fig. 9d), and ROC curve analysis showed that the AUC of the model was 0.78 (Fig. 9e).

### Validation of the expression levels of the 9 MRGs in clinical samples

**Fig. 10:**
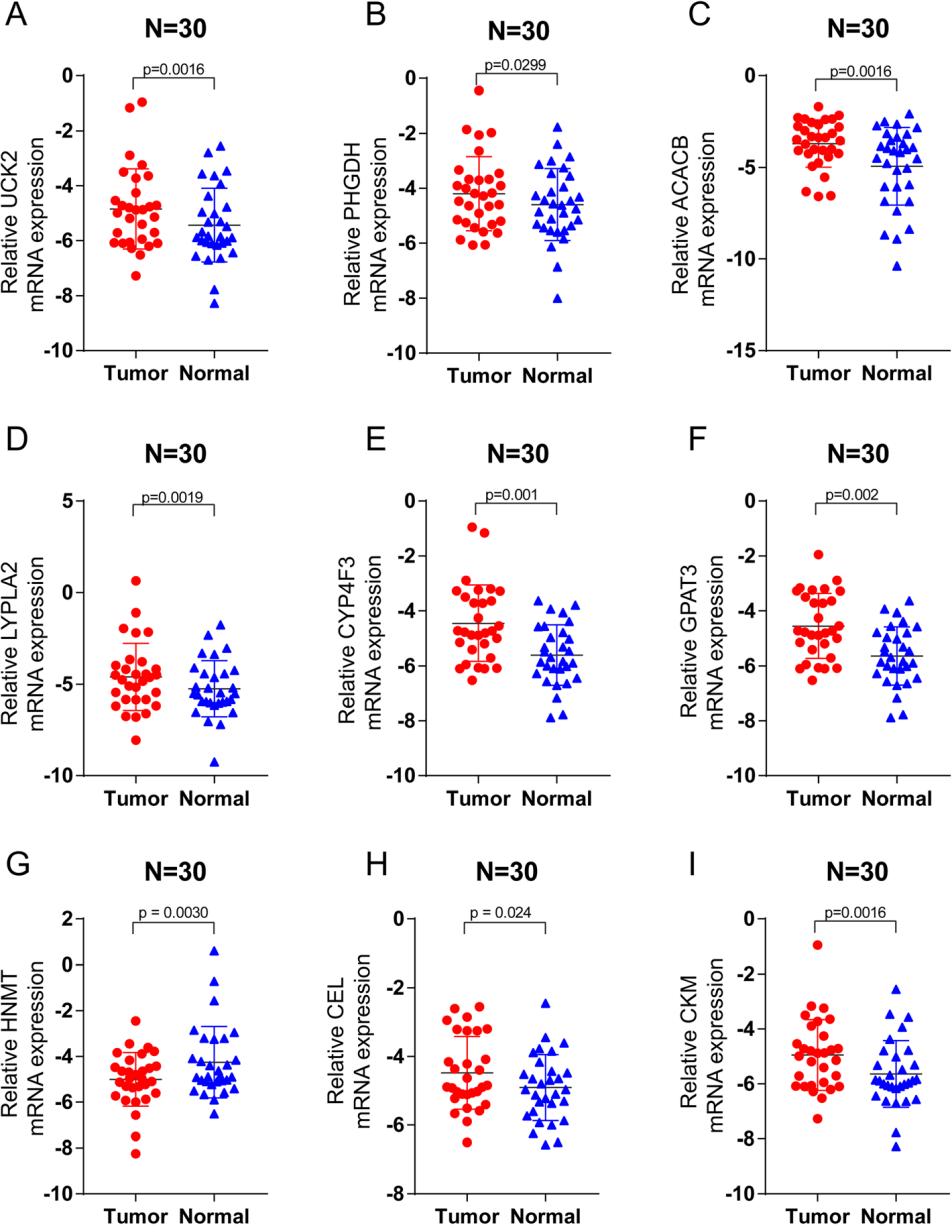
Validation of the expression signature of 9 MRGs in tissues by qRT-PCR. Student’s t-test (two-tailed) was used for the comparison analyses and the significance threshold was set at 0.05 for in each test

The expression signatures of the 9 MRGs were subsequently explored in 30 endometrial cancer clinical specimens. The results demonstrated that UCK2, PHGDH, ACACB, LYPLA2, CYP4F3, GPAT3, CEL, and CKM mRNA level were upregulated in cancerous tissues, while HNMT was downregulated, which was in accordance with the above findings (Fig. 10a-i).

### The clinical independence and correlation estimation of the risk signature

**Fig. 11:**
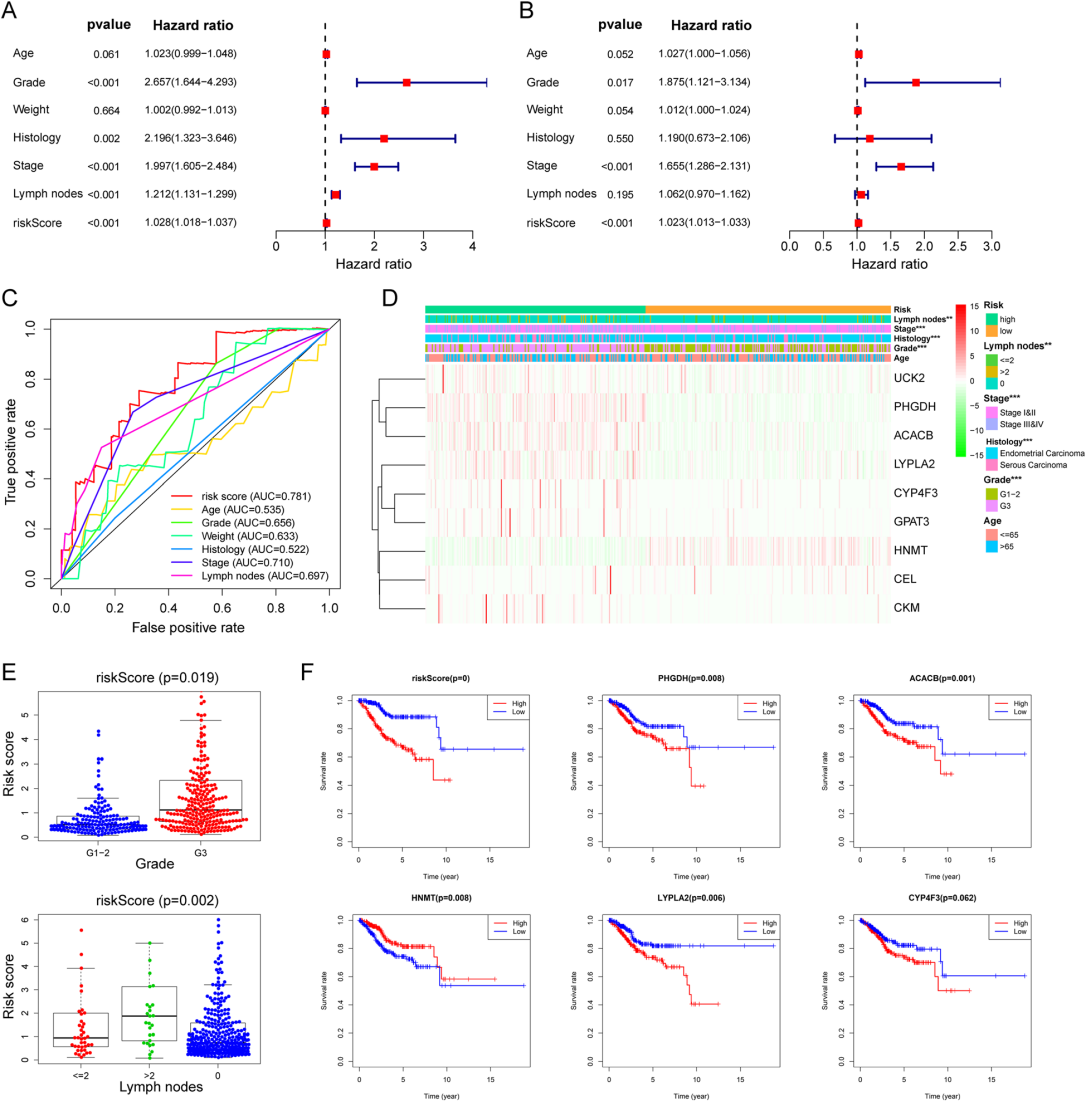
Clinical characteristics of the prognostic MRGs signature. Univariate (**a**) and multivariate (**b**) Cox regression analysis, as well as time-dependent ROC curve analysis (**c**) of the prognostic value between the risk model and EC patients’ OS status when compared to or combined with clinical factors; **d** Heat map showing the expression of 9 MRGs in the risk model and the clinicopathological features of patients with EC; **e** Clinicopathological significance of the prognostic signature of endometrial cancer; **f** Kaplan-Meier curve plot of the prognostic MRGs from the signature

Then, we combined the risk model with other clinical factors and performed univariate and multivariate analyses to examine the clinical independence of the model. The results showed that the model was able to serve as an independent prognostic indicator (both *P* < 0.001) (Fig. 11a-b). The AUC value of the prognostic model was 0.781, which was significantly higher than that of patients’ age (AUC = 0.535) and weight (AUC = 0.633), clinical-stage (AUC = 0.710), tumor grade (AUC = 0.656), histology (AUC = 0.522), and lymph node status (AUC = 0.697) (Fig. 11c). Next, we assessed the risk scores, clinical features, and nine-gene expression profiles of EC patients and displayed them in the heatmap shown in Fig. 11d. Interestingly, the clinical characteristics of the patients were highly in accordance with the risk level calculated from the model. The high-risk subgroup patients were characterized by late-stage, high-grade, serous carcinoma, and more metastatic lymph nodes (Fig. 11d-e), which all presented worse outcomes. The correlations between each gene from the prognostic model and the patients’ clinical features were also measured. PHGDH, ACACB, HNMT, CYP4F3, and LYPLA2 were shown to be significantly associated with patient prognosis (Fig. 11f). The other clinical features of each prognostic MRG from the signature are presented in Supplementary Fig. 3.

### Nomogram building and validation

**Fig. 12:**
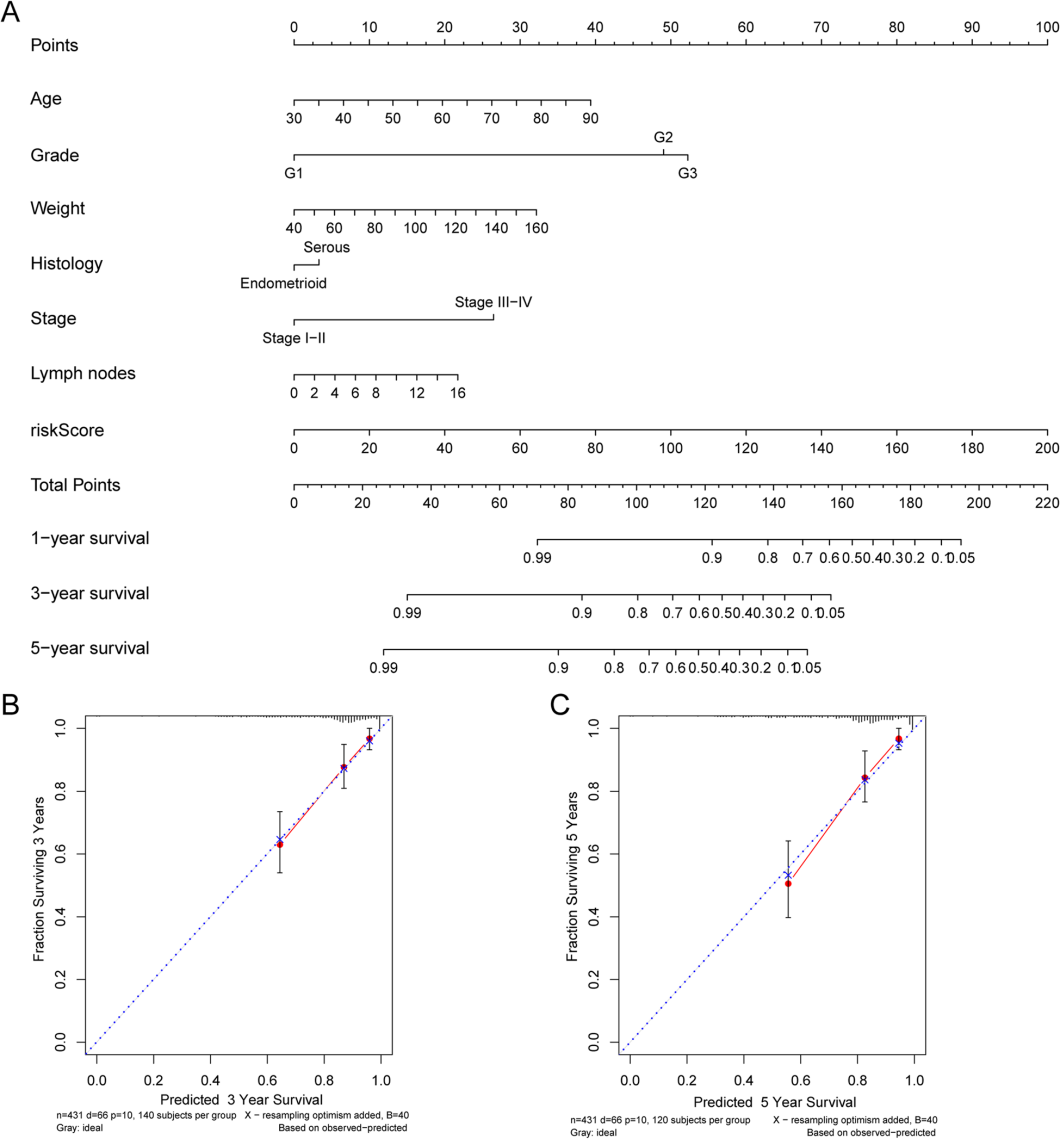
Nomogram for predicting the 5-year survival probability of patients with EC. **a** Prognostic nomogram for EC patients; **b-c** Calibration curves for the nomogram at (**b**) 3-, and (**c**) 5-year

Based on the patients’ risk scores and clinical features, we built a comprehensive prognostic nomogram to estimate EC patients’ survival probability for 5 years based on the entire TCGA set. Seven independent prognostic parameters, including metabolic risk signature, age, grade, weight, histology, stage and lymph node status, were integrated into the nomogram (Fig. 12a). The calibration plots showed excellent consistency between the nomogram predictions and actual observations in terms of the 3- and 5-year survival rates in the TCGA cohort (Fig. 12b-c).

## Discussion

Metabolic abnormalities have recently been widely studied and shown to play an important role in tumor development in various cancer types. Metabolic dysfunction in the tumor microenvironment could lead to various outcomes in patients, and metabolism-related genes can be used as prognostic markers of tumors. In this work, we thoroughly investigated the implications of metabolism-related genes in endometrial cancer progression. By analyzing the mRNA data of TCGA EC patients, we obtained 220 dysregulated MRGs, among which 47 were associated with EC patients’ OS. Functional enrichment analysis of these prognostic MRGs showed that they were closely associated with the cellular amino acid metabolic process, glycolysis, and glycerophospholipid metabolism. In accordance with our observation, Byrne et al. also found that glycolysis and lipogenesis are highly associated with endometrial cancer phenotypes and that the suppression of GLUT6 gene expression could inhibit glycolysis and the survival of EC cells, underlying the crucial role of energy metabolism in tumor progression [16]. In addition, our results further revealed the exact dysregulated metabolic genes of these disordered metabolism-related pathways, which may provide a new perspective on the molecular mechanisms of metabolism alterations in tumor progression.

Metabolic prognostic risk signatures that combined the expression of multiple metabolism-related genes have been indicated to serve as powerful prognostic indicators in various malignant diseases, such as glioma, liver cancer, ovarian cancer, and papillary thyroid carcinoma. Zhou et al. identified a 29-energy metabolism-related gene signature, containing branched-chain amino acid transaminase 1 (BCAT1), interleukin-4 and carbohydrate sulfotransferases, to evaluate the prognosis of diffuse glioma [17]. Wang et al. enrolled 6 risks and 2 protective metabolic genes into the prognostic metabolic model which effectively predicted ovarian cancer patients’ prognosis [18]. Likewise, Ma et al. developed a metabolic gene signature as a biomarker for dedifferentiated thyroid cancer [19], and Liu et al. built a four-metabolic gene signature for liver cancer patient outcome prediction [20].

In the present study, we performed LASSO and multivariate Cox regression analyses and identified a nine-gene signature including CYP4F3, CEL, GPAT3, LYPLA2, HNMT, PHGDH, CKM, UCK2, and ACACB. Among them, HNMT was considered a protective factor while others were risk factors. The diagnostic and predictive effectiveness of these prognostic genes has already been reported in other studies. Cui et al. reported a significantly higher expression of carboxyl ester lipase (CEL) in breast cancer. The combination of CEL and other biomarkers could improve the diagnostic capability for breast cancer [21]. Likewise, Richard et al. found that over 70% of estrogen receptor (ER)-negative breast cancers exhibited elevated phosphoglycerate dehydrogenase (PHGDH) protein expression, which is crucial for promoting serine pathway flux [22]. Li reported downregulation of PHGDH caused by overexpressing LncRNA PlncRNA-1 mediated cell apoptosis rate in breast cancer [23]. In addition, Zhang et al. discovered that PHGDH could define a metabolic subtype in lung adenocarcinomas with unique metabolic dependencies [24]. In pancreatic ductal adenocarcinoma, CYP4F3, one isoform of the cytochrome P450 (CYP) superfamily, was shown to be upregulated in tumor tissues and could serve as a distinguishing marker [25]. Uridine-cytidine kinase 2(UCK2) was positively correlated with early recurrence and poor prognosis in hepatocellular carcinoma. Overexpression of UCK2 increased MMP2/9 expression and further activated Stat3 signaling, mediating the metastasis of hepatocellular carcinoma cells [26]. For ACACB, Lally et al. showed that humans with fatal HCC subtypes have increased acetyl-CoA carboxylase (ACC) expression and that the genetic activation of ACC promoted the formation of hepatic de novo lipids and induced subsequent liver carcinogenesis [27].

Here, through bioinformatic analysis and outside validation, we innovatively reported that these metabolic genes are closely related to the prognosis of EC patients. In addition, the metabolic risk signature combining these genes could accurately categorize EC patients into high- or low-risk subgroups which represented patients’ long-term outcomes. Last, our study was the first to build a comprehensive nomogram that incorporated a metabolism-related signature with clinical features including age, stage, tumor grade and lymph node status to effectively predict the survival of EC patients. This prognostic scoring system could provide a precise method to help both physicians and patients perform individualized survival evaluations and select treatment options.

## Conclusion

In conclusion, we identified 47 prognosis-related dysregulated metabolic genes in EC. The prognostic DE-MRGs were highly associated with amino acid, glycolysis, and glycerophospholipid metabolism. The top 15 hub genes in the PPI network were also identified and analyzed. We performed LASSO and multivariate Cox regression analyses to establish and validate a robust prognostic risk signature enrolling the nine dysregulated MRGs. In addition, a comprehensive nomogram that combined clinical characteristics and the risk model was constructed, and its efficacy in predicting EC patients’ prognosis was also demonstrated. The 9-MRG model and nomogram may guide the selection of rational therapeutic strategies for doctors in clinical practice.

## Supplementary information

**Additional file 1: Supplementary Table 1.** The gene list of all metabolic genes.

**Additional file 2: Supplementary Table 2.** qRT-PCR Primers for nine metabolic genes.

**Additional file 3: Supplementary Fig. 1.** GO and KEGG pathway enrichment of 220 DE-MRGs.

**Additional file 4: Supplementary Fig. 2.** The protein-protein interaction network of 47 DE-MRGs.

**Additional file 5: Supplementary Fig. 3.** Clinical characteristics of each prognostic MRG from the signature.

## Data Availability

The expression data were deposited in the TCGA database and the clinical information was retrieved from the cBioPortal website. Besides, please contact the author for data and materials requests.

## Abbreviations

EC: Endometrial cancer
MRGs: Metabolism-related genes
TCGA: The Cancer Genome Atlas
GO: Gene ontology
KEGG: Kyoto Encyclopedia of Genes and Genomes
PPI: Protein-protein interaction
STRING: Search Tool for the Retrieval of Interacting Genes Database
ROC: Receiver operating characteristic
AUC: Area under the curve
LASSO: Least Absolute Shrinkage and Selection Operator
OS: Overall survival
FC: Fold change
