# Supplementary material for: Identification of a metabolism-related gene expression prognostic model in endometrial carcinoma patients
Source: BMC Cancer. 2020 Sep 7;20:864. doi: 10.1186/s12885-020-07345-8 (PMC7487491; doi:10.1186/s12885-020-07345-8)
Supplement: Supplementary file 1 — Additional file 1: Supplementary Table 1. The gene list of all metabolic genes. [file 12885_2020_7345_MOESM1_ESM.docx]

| **Supplementary Table 1 The gene list of all metabolic genes** |
| --- |

AKR1C4

PRODH

NME6

GK

CKMT1A

PAICS

CES1

TYMP

GLS2

POLR2D

SAT1

UAP1

DGKG

UPB1

AKR1B10

TH

POLD4

PLA2G5

LIPF

ACAT1

UGT2B7

PIP4K2C

NT5C

ARG1

DNMT3L

DHRS4L2

RDH10

PNLIPRP1

CES2

ADH1B

CMPK2

UGT2B17

ADCY9

PGM2L1

CNDP1

GSTZ1

CPOX

ENPP7

BST1

ACOX3

GSTA3

ENTPD6

HMGCS2

GNPAT

ALOX12B

MCEE

AOC2

PFKL

SGPL1

ACP4

PLCD3

DGKZ

FECH

POLR3B

SULT1A4

PIKFYVE

PLA2G10

GDA

GPX3

GPD2

CYP26B1

LCLAT1

PDE1A

CA8

ENPP1

PLCE1

GMPR2

MIOX

PNLIP

NOS2

NT5C2

GK2

POLR2A

UPP1

ACP2

AKR1C3

GANC

GSTK1

MTR

GSS

ALDH1A1

APIP

ENTPD1

HK2

POLR2F

GSTA2

BAAT

CYP2A6

AACS

UGT2A3

CA13

TREH

CYB5R3

BLVRB

PLA2G12B

COMT

NNT

ADSS

PDE6D

TYMS

GSTM2

PDHB

UGT2B4

NAGK

UGT1A7

NMNAT1

CYP26A1

NME4

CEL

UGT1A9

METTL6

PKLR

CDS2

GOT1

FTMT

POLD3

MAOB

TAT

B4GALT2

GRHPR

ECI2

NANP

LCT

PAPSS1

AGMAT

MTHFD2L

PLD1

AMY1C

PDE1C

UMPS

AKR1B1

UCKL1

AKR1A1

GCLC

OXCT1

UGT1A10

GLA

CYP1A2

ITPKB

LDHAL6A

PISD

PDE5A

XDH

AGL

ALDH3A1

IMPDH2

NOS1

TSTA3

PIK3CA

ALDH9A1

UGDH

PLCB1

RPE65

GUSB

CYP4F2

ARSA

PHOSPHO1

IMPDH1

MPST

CYP2B6

DCT

NMRK1

SYNJ2

ADH5

PDE8B

UGT1A4

ALDOA

UCK2

ANPEP

PDE6C

DHODH

G6PC2

MTHFD2

TK1

HADH

AOX1

PFKM

AK1

INPP5K

CA5B

LAP3

HGD

PPOX

POLR3D

NT5E

HMBS

PLA2G4B

TRMT11

ALOX15B

HEXA

GLB1

ADH4

GALK2

NUDT5

TXNRD1

GYS2

GPT2

UGT2B10

SGPP2

LDHB

ITPKA

PDHA2

CYB5R1

NAT1

NME1-NME2

PYGL

ALDH2

PGM2

NMNAT3

INPPL1

CPT1B

PDE6A

GMPR

ALDH4A1

ACAT2

CBR3

PC

TRDMT1

TXNRD2

PLCB4

GMPPA

SUCLG2

ALDH3B1

GLUD1

PIP5K1A

AMT

AOC1

ITPK1

PCYT1A

DEGS2

ALDH5A1

PIK3CD

SULT2B1

GLS

CDS1

PGM1

ACADS

INPP4A

HK3

ACAA1

DHRS3

PYCR3

IMPA1

MPI

ADCY2

GLYCTK

IDO1

PDE4A

PTGES2

ETNK1

INPP4B

PLCB2

LDHAL6B

GPAT3

SMS

DPYD

PDE6B

HYI

PTDSS1

HMGCL

ASPA

ACADVL

IPMK

ENTPD3

GUCY1B1

DHRS4

PRPS1L1

POLD2

WARS2

RRM2

ALDH3B2

GALE

ACER1

CBS

ACPP

GGT5

AGPAT3

G6PD

HADHA

ACSM3

ITPA

ASNS

CHST11

CA5A

MARS2

AGPS

ADH7

PRODH2

NADK

GCLM

CYP2C18

ADO

DEGS1

PSPH

DMGDH

LTC4S

ADCY1

CYP2C19

HAGHL

DCK

CPT2

APRT

CAD

ALAS2

FLAD1

PLA2G2F

PIK3C3

PDE7A

PDHA1

FPGT

HPD

ACSM2A

CYP4A22

RDH8

OAT

PLPP3

GUCY1A1

MGLL

AGK

ACO1

DPYS

RDH16

DGAT2

PAFAH1B1

ARG2

HEMK1

ME2

MTAP

PHGDH

UROS

CKMT2

MINPP1

GPD1L

PDE4D

ACSL1

DHDH

GGT7

LTA4H

CYP4A11

PYCR2

TPH1

CA1

CHKB

ACY1

GNPNAT1

ME3

UGT2A1

PMM2

GALK1

POLR3K

GPD1

GPX2

CHST12

IMPA2

POLR2K

AMD1

PSAT1

ENTPD5

NPL

DAO

DTYMK

PGP

TKFC

CYP26C1

NAA80

CA12

AFMID

PLA2G12A

ASAH1

LPCAT3

CA7

DGKA

MAOA

BUD23

PTGIS

LPGAT1

EHHADH

FMO1

UGT1A8

CYP3A7

PDE1B

ASMT

PDE3A

ADSSL1

AMY1A

ADCY5

DUT

GAD1

INPP5E

CA14

ALOX5

MTMR2

AHCYL2

PTGS1

SGMS1

OPLAH

BLVRA

NEU4

DGKQ

HAGH

PYCR1

SMPD1

GSTM1

PTGS2

CYP3A5

MAT2A

PNMT

CTPS1

GGT1

ACER3

AHCYL1

POLR3H

PLCB3

GPAM

CHST13

RFK

SEPHS2

PLA2G4A

CPT1A

GMPS

DCTD

PLA2G4E

SARDH

GGT6

GAMT

FTH1

AK3

ECHS1

CYP1A1

CKMT1B

HPGDS

FHIT

PAFAH2

CYP3A4

GSTO1

UPRT

TDO2

METTL2B

PCK1

ALDOB

CHAT

ZNRD1

GLO1

INPP5B

HPRT1

PFKFB2

POLR1E

ALAS1

PDE7B

GYS1

ENPP6

AGPAT4

PLA2G1B

ACO2

LDHA

TYRP1

GAD2

GPT

MARS

LYPLA2

GSTP1

DLAT

AGXT2

ALAD

TXNDC12

GBE1

GNE

FMO4

DGKB

CYP2J2

HMOX2

ACYP2

GNPDA2

DGUOK

ENOPH1

NADSYN1

ALOX12

PNPLA4

ALDH1A3

ENPP3

PRUNE1

MMAB

PLCD4

ENTPD4

LCMT2

NPR2

SUCLG2P2

DDC

CHIT1

CHDH

NME2

PLA2G2E

PRIM1

CHPT1

ALDH1B1

HAO1

POLR2J2

SHMT1

POLR1C

PDE3B

RRM2B

ACMSD

TBXAS1

GART

PDE4B

GUCY2C

CRLS1

DNMT1

SORD

ME1

UROC1

FMO5

POLE3

GLUL

PIK3C2B

GPI

BCO1

PLCD1

HNMT

HAL

URAD

ACACB

POLR2C

GSTM5

GALT

ACP1

PIPOX

GBA

NOS3

PMM1

PLD2

GPAT2

UCK1

ALDH6A1

AANAT

SRM

GSTM3

TYR

GSTT2

CERK

PCCA

AKR1C1

DGKI

CYP2D6

POLD1

ADH6

NUDT2

HAAO

PKM

NPR1

DGKD

GPX4

UGT2B11

POLR2H

PRIM2

AMPD1

POLE4

NEU3

NT5C3A

CYP2U1

HK1

CDIPT

IDO2

NUDT9

GSTA4

GUK1

CANT1

PIP4K2A

B4GALT1

MBOAT7

PCYT1B

SMPD3

ACYP1

EPHX2

MBOAT2

CTPS2

BPNT1

PLA2G15

LIPG

AOC3

NT5M

PGS1

FBP2

SMPD2

PDE11A

PI4KB

NNMT

PDE9A

SI

PCYT2

PDE2A

ACY3

OGDHL

ADSL

GMPPB

AMY2B

AK7

CDO1

FMO2

TK2

FTCD

PFKP

INPP1

CAT

GUCY2F

MGST1

LRAT

PLA2G6

DNMT3A

P4HA1

WARS

LDHD

ACSL3

UGT1A5

SPHK1

LALBA

EARS2

PYGM

PTEN

LCMT1

GATM

PCK2

KMO

ODC1

GCK

HMGCS1

POLR2J

AMY1B

CS

PTGES

GSTO2

NMNAT2

AK5

AHCY

OXCT2

POLR3C

AK4

ENTPD8

CKB

G6PC

PTDSS2

AMDHD2

GGCT

NUDT12

HMOX1

UROD

CA6

SDS

BDH1

OTC

IDH2

ACSS2

RDH5

ACADL

MTMR7

HADHB

ALDH1A2

PI4KA

LPCAT4

CPS1

ISYNA1

ETNK2

PTGDS

MLYCD

SUOX

TPI1

GLDC

CSAD

GALC

IPPK

B4GALT6

PNP

GUCY1A2

ADCY3

INPP5A

PIP5K1C

SRR

PLCG2

DDO

P4HA2

POLR1A

NAMPT

JMJD7-PLA2G4B

CYP2F1

PRPS1

CES5A

SGMS2

PEMT

SUCLA2

PAFAH1B2

RDH12

CYP2S1

PCCB

ADI1

SCLY

PAH

PRPS2

POLR1B

ACAA2

NME7

CA9

PDE10A

DHRS9

NT5C1A

ADH1A

AGPAT1

ADCY6

ENPP2

PLA2G2C

EPHX1

SMPD4

RDH11

UXS1

GSTA5

LIPC

LCAT

GPX7

GMDS

ACADSB

RETSAT

ACSS1

ABAT

HCCS

MGAM

POLR3GL

MTHFD1L

MTMR1

EPRS

COX15

CYP2C9

ACACA

PLCZ1

CYP1B1

GCAT

GNPDA1

DLD

LYPLA1

ACHE

BHMT

ADCY10

TAZ

CD38

NEU1

POLR2B

SULT1A1

CKM

FAH

INMT

AKR1C2

SULT1E1

GFPT1

CHKA

GLUD2

ASS1

PLA2G2A

P4HA3

GAA

AZIN2

ADK

MTHFD1

ACER2

PIK3CG

ACP5

AGPAT2

GCDH

MGST3

NIT2

AMPD2

GUCY2D

QPRT

POLA2

SEPHS1

UGP2

ACSL4

BDH2

NEU2

ALDOC

PIP5K1B

PLPP2

POLR2J3

ACP6

RRM1

GSTM4

TPMT

GBA3

IDH1

ASAH2

PLA2G2D

LPCAT2

ALDH18A1

ACSL6

IL4I1

GSTA1

LDHC

PIK3C2A

SGPP1

ACSL5

ACSS3

HDC

POLE

CHIA

PIK3CB

PHPT1

ACSM4

COX10

PNPLA3

OGDH

CMPK1

SAT2

CYP2C8

PYGB

ADCY4

CA2

NANS

CDA

GFPT2

ACOT12

AMDHD1

MAT2B

MBOAT1

AMPD3

PFKFB4

DBH

GOT2

MDH1

LPCAT1

SULT1A3

ADCY8

MDH2

HEXB

NAGS

CYP4F3

PGD

AMY2A

PNLIPRP2

HIBCH

GNMT

PLA2G7

PFKFB1

ALLC

GPX5

CA3

KHK

HAO2

POLR1D

SPHK2

ACSM1

ACOX1

POLR3F

DGKE

LPL

CA4

PFKFB3

PIP4K2B

SUCLG1

PGM3

DGKH

UGT8

SPTLC1

NAT2

PRDX6

CTH

POLE2

PNPT1

SHMT2

PLA2G3

GAL3ST1

UGT2B28

CYP3A43

CBR1

ATIC

ENTPD2

DGAT1

PLPP1

GSR

MIF

ADCY7

INPP5J

CMAS

AWAT2

AC005759.1

GPX6

PIK3C2G

NT5C1B

AGXT

GPAT4

DNMT3B

MGST2

POLR3G

UGCG

PFAS

POLR2I

OCRL

AC139530.1

ACSM5

TPO

L2HGDH

FBP1

RENBP

ADH1C

AADAT

PAPSS2

MAT1A

SYNJ1

ADPRM

GPX1

AK2

ALDH3A2

FMO3

CP

UGT1A3

PLCG1

TPH2

CYP2E1

UPP2

PDE8A

CYP2A13

POLA1

POLR3A

PPAT

PAFAH1B3

POLR2L

POLR2E

SPTLC2

NME3

NME5

SULT1A2

CPT1C

FADS2

ADA

ACADM

ALDH7A1

ECI1

UGT1A1

UGT2B15

ASL

UGT1A6

CYP2A7

MTMR6

KYNU

ALOX15

NME1

KDSR

POLR2G
