# Supplementary material for: Identification of a metabolism-related gene expression prognostic model in endometrial carcinoma patients
Source: BMC Cancer. 2020 Sep 7;20:864. doi: 10.1186/s12885-020-07345-8 (PMC7487491; doi:10.1186/s12885-020-07345-8)
Supplement: Supplementary file 2 — Additional file 2: Supplementary Table 2. qRT-PCR Primers for nine metabolic genes. [file 12885_2020_7345_MOESM2_ESM.docx]

| **Supplementary Table 2 qRT-PCR Primers for nine metabolic genes** | | |
| --- | --- | --- |
| CYP4F3 | Forward Primer | CAACCCCCGAAACGGAATTG |
|  | Reverse Primer | TTCCTCCGAGCTGTGAATCAG |
| CEL | Forward Primer | TGGGTGACTCTGTGGACATCT |
|  | Reverse Primer | GCAGGCATCTCTTCTTGAAGTT |
| GPAT3 | Forward Primer | GAGGGCCTCCAGGTGAGT |
|  | Reverse Primer | CTCAGGAGAGGTCCGCAGT |
| LYPLA2 | Forward Primer | CCCTCACGTCAAGTACATCTGT |
|  | Reverse Primer | GACGATTCGATTGGCAGGGAT |
| HNMT | Forward Primer | GGAGGTGCAGATTGTCTCATTC |
|  | Reverse Primer | TCATGCCTGGCTTGTCTTTCT |
| PHGDH | Forward Primer | CTGCGGAAAGTGCTCATCAGT |
|  | Reverse Primer | TGGCAGAGCGAACAATAAGGC |
| CKM | Forward Primer | ATGCCATTCGGTAACACCCAC |
|  | Reverse Primer | GCTTCTTGTAGAGTTCAAGGGTC |
| UCK2 | Forward Primer | GCCCTTCCTTATAGGCGTCAG |
|  | Reverse Primer | CTTCTGGCGATAGTCCACCTC |
| ACACB | Forward Primer | CAAGCCGATCACCAAGAGTAAA |
|  | Reverse Primer | CCCTGAGTTATCAGAGGCTGG |
