# Supplementary figures and images for: Identification of a metabolism-related gene expression prognostic model in endometrial carcinoma patients
Source: BMC Cancer. 2020 Sep 7;20:864. doi: 10.1186/s12885-020-07345-8 (PMC7487491; doi:10.1186/s12885-020-07345-8)

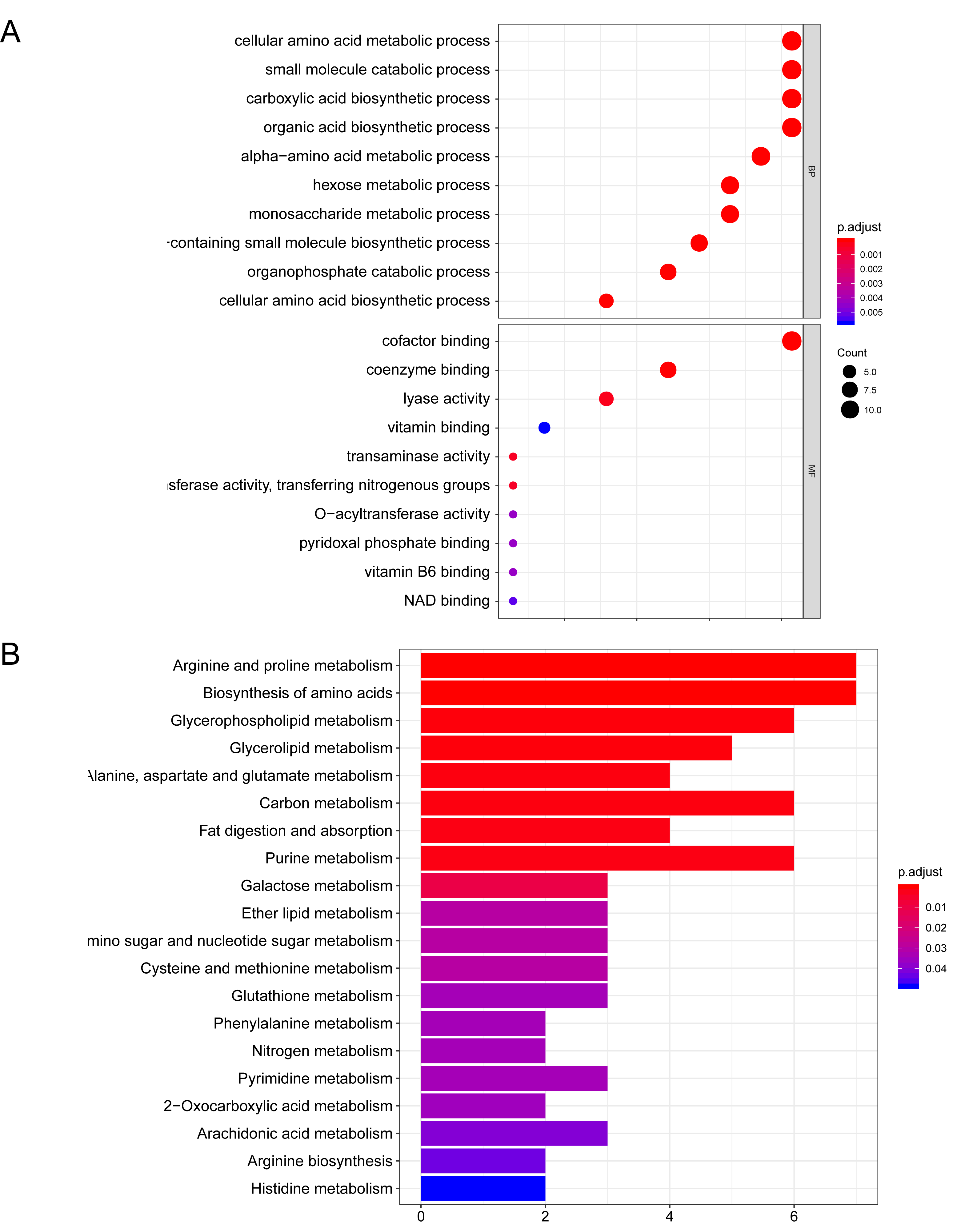

Supplement: Supplementary file 3 — Additional file 3: Supplementary Fig. 1. GO and KEGG pathway enrichment of 220 DE-MRGs. [file 12885_2020_7345_MOESM3_ESM.tif]

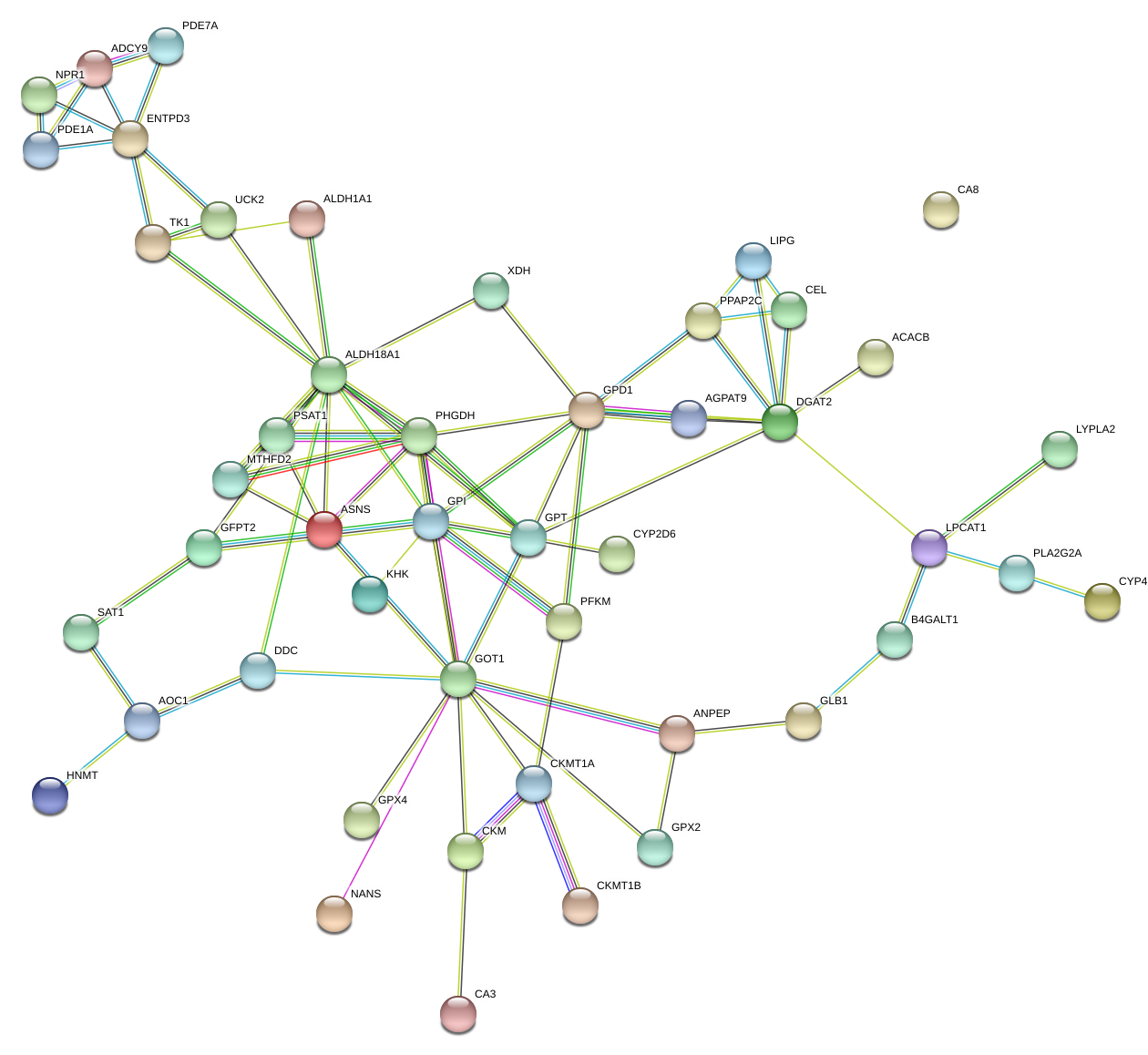

Supplement: Supplementary file 4 — Additional file 4: Supplementary Fig. 2. The protein-protein interaction network of 47 DE-MRGs. [file 12885_2020_7345_MOESM4_ESM.png]

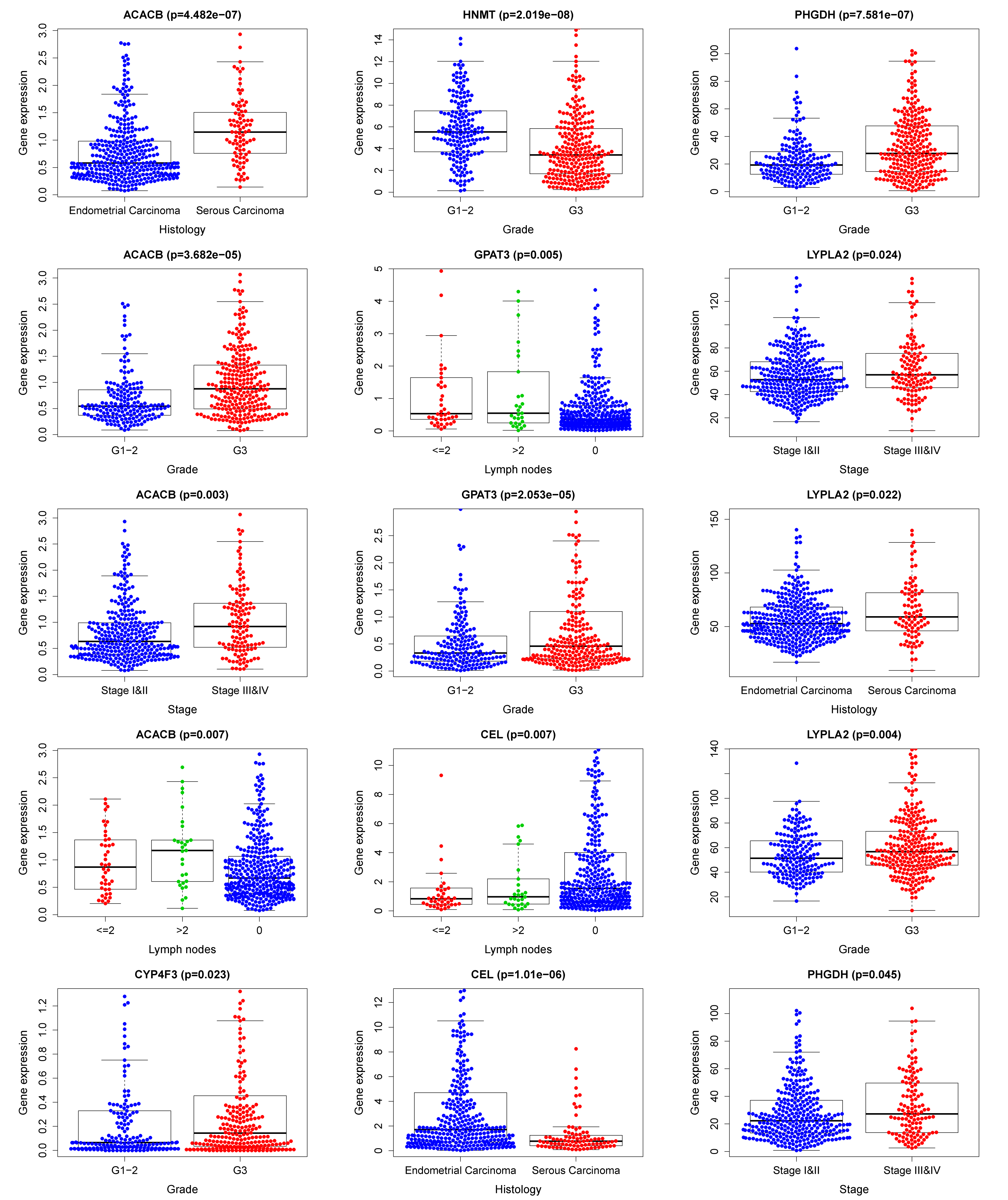

Supplement: Supplementary file 5 — Additional file 5: Supplementary Fig. 3. Clinical characteristics of each prognostic MRG from the signature. [file 12885_2020_7345_MOESM5_ESM.tif]
